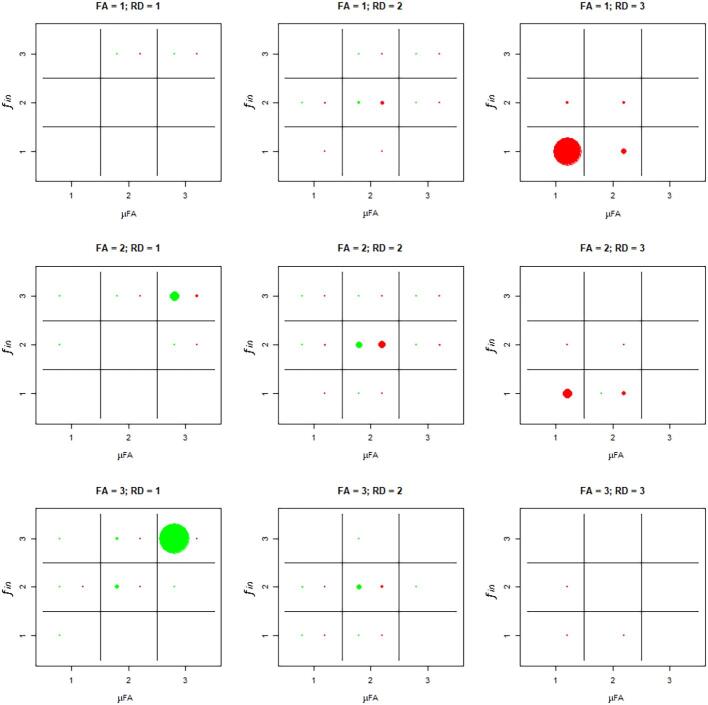# Characterization of multiple sclerosis lesions with distinct clinical correlates through quantitative diffusion MRI

**DOI:** 10.1016/j.nicl.2020.102411

**Published:** 2020-09-09

**Authors:** Eloy Martínez-Heras, Elisabeth Solana, Ferran Prados, Magí Andorrà, Aleix Solanes, Elisabet López-Soley, Carmen Montejo, Irene Pulido-Valdeolivas, Salut Alba-Arbalat, Nuria Sola-Valls, Maria Sepúlveda, Yolanda Blanco, Albert Saiz, Joaquim Radua, Sara Llufriu

**Affiliations:** a Center of Neuroimmunology, Laboratory of Advanced Imaging in Neuroimmunological Diseases, Hospital Clinic Barcelona, Institut d’Investigacions Biomèdiques August Pi i Sunyer (IDIBAPS) and Universitat de Barcelona, Barcelona, Spain; b E-health Centre, Universitat Oberta de Catalunya, Barcelona, Spain; c Centre for Medical Image Computing (CMIC), Department of Medical Physics and Bioengineering, University College London, London, UK; d NMR Research Unit, Queen Square MS Centre, Department of Neuroinflammation, UCL Institute of Neurology, University College London, London, UK; e Imaging of Mood- and Anxiety-related Disorders (IMARD) Group, IDIBAPS and CIBERSAM, Barcelona, Spain; f Centre for Psychiatry Research, Department of Clinical Neuroscience, Karolinska Institutet, Stockholm, Sweden; g Early Psychosis: Interventions and Clinical-detection (EPIC) Lab, Department of Psychosis Studies, Institute of Psychiatry, Psychology and Neuroscience, King's College London, London, UK

**Keywords:** T1 3D-MPRAGE, 3D-Magnetization Prepared Rapid Acquisition Gradient Echo, 3D-T2, 3D-T2 fluid-attenuated inversion recovery, AD, Axial diffusivity, BRB-N, Brief Repeatable Battery of neuropsychological tests, DTI, Diffusion tensor imaging, DWIs, Diffusion-weighted images, EDSS, Expanded disability status scale, *v*_MD_, Extra-neurite microscopic mean diffusivity, *v*_AD_, Extra-neurite transverse microscopic diffusivity, FA, Fractional anisotropy, ƒ_in_, Intra-neurite volume fraction, λ_diff_, Intrinsic diffusivity, MC-SMT, Multi-compartment spherical mean technique, MD, Mean diffusivity, MS, Multiple sclerosis, MSSS, Multiple sclerosis severity score, NAWM, Normal-appearing white matter, RD, radial diffusivity, RR, Relapsing remitting, SP, Secondary progressive, MRI, Diffusion magnetic resonance imaging, K-means clustering algorithm, MS lesion types, Multiple Sclerosis

## Abstract

Diffusion magnetic resonance imaging can reveal quantitative information about the tissue changes in multiple sclerosis. The recently developed multi-compartment spherical mean technique can map different microscopic properties based only on local diffusion signals, and it may provide specific information on the underlying microstructural modifications that arise in multiple sclerosis. Given that the lesions in multiple sclerosis may reflect different degrees of damage, we hypothesized that quantitative diffusion maps may help characterize the severity of lesions “*in vivo*” and correlate these to an individual’s clinical profile. We evaluated this in a cohort of 59 multiple sclerosis patients (62% female, mean age 44.7 years), for whom demographic and disease information was obtained, and who underwent a comprehensive physical and cognitive evaluation. The magnetic resonance imaging protocol included conventional sequences to define focal lesions, and multi-shell diffusion imaging was used with b-values of 1000, 2000 and 3000 s/mm^2^ in 180 encoding directions. Quantitative diffusion properties on a macro- and micro-scale were used to discriminate distinct types of lesions through a k-means clustering algorithm, and the number and volume of those lesion types were correlated with parameters of the disease. The combination of diffusion tensor imaging metrics (fractional anisotropy and radial diffusivity) and multi-compartment spherical mean technique values (microscopic fractional anisotropy and intra-neurite volume fraction) differentiated two type of lesions, with a prediction strength of 0.931. The B-type lesions had larger diffusion changes compared to the A-type lesions, irrespective of their location (P < 0.001). The number of A and B type lesions was similar, although in juxtacortical areas B-type lesions predominated (60%, P < 0.001). Also, the percentage of B-type lesion volume was higher (64%, P < 0.001), indicating that these lesions were larger. The number and volume of B-type lesions was related to the severity of disease evolution, clinical disability and cognitive decline (P = 0.004, Bonferroni correction). Specifically, more and larger B-type lesions were correlated with a worse Multiple Sclerosis Severity Score, cerebellar function and cognitive performance. Thus, by combining several microscopic and macroscopic diffusion properties, the severity of damage within focal lesions can be characterized, further contributing to our understanding of the mechanisms that drive disease evolution. Accordingly, the classification of lesion types has the potential to permit more specific and better-targeted treatment of patients with multiple sclerosis.

## Highlights

• Macroscopic and microscopic diffusion properties discriminate between MS lesion types.
• The number and volume of lesions with larger diffusion changes are associated with worse clinical outcomes.
• Diffusion MRI provides useful information of the pathological heterogeneity in plaques.

## 1 Introduction

Multiple sclerosis (MS) is a chronic inflammatory autoimmune disease of the central nervous system (CNS) that is characterised by the presence of focal lesions, and damage to the normal-appearing white matter (NAWM) and the grey matter (Lassmann et al., 2007). There is substantial heterogeneity in the pathological changes among MS lesions, with different patterns of demyelination (Lucchinetti et al., 2000) and a variable degree of neuroaxonal damage having been described (Ludwin, 2006). In addition, while active plaques are most often found at early disease stages, smoldering, inactive and shadow plaques subsequently predominate. Chronic active lesions are associated with a more aggressive disease evolution (Absinta et al., 2019, Lucchinetti et al., 2000) and indeed, differences in the severity of demyelination, remyelination and neuroaxonal damage could explain why some patients recover completely from relapses yet in others, their disability deteriorates more rapidly.

The changes in lesions and in the NAWM can be visualised through conventional magnetic resonance imaging (MRI), yet they are poorly associated with the clinical phenotype and physical disability (Barkhof, 2002), partly reflecting the failure to characterise the pathological nature of tissue injury in MS. However, diffusion MRI-based techniques can reveal quantitative and more specific information about the mechanisms associated with tissue changes (Rocca et al., 2015). Macroscopic diffusion properties have been studied extensively in MS lesions using diffusion tensor imaging (DTI) features, such as the reduction in fractional anisotropy (FA) relative to the NAWM. Unfortunately, DTI findings are strongly influenced by a complex intravoxel fibre architecture, which limits the ability to accurately estimate the different pathophysiological features of the disease (Rovaris et al., 2005, Filippi and Rocca, 2011).

Recently, several microstructure imaging techniques have been proposed to compute distinct signal contribution patterns with the aim to provide greater sensitivity and specificity toward the underlying damage mechanisms (Novikov et al., 2019). Several mathematical representations from biophysical models have been exploited to understand the contribution of restricted intracellular diffusion components (Kroenke et al., 2004). The estimation of local diffusion properties based on multi-compartment spherical mean technique (MC-SMT) has successfully decomposed the distinct signal components into microscopic tissue features (Kaden et al., 2016). Thus, this approach is only sensitive to fibre composition, whereas DTI metrics depend on both intravoxel fibre orientation, distribution and microstructure (Mollink et al., 2017, Jones et al., 2018). The MC-SMT model computes a multi-compartment domain, encompassing extra-axonal and intra-axonal water diffusion spaces, and microscopic diffusion tensor maps to estimate distinct local tissue properties (Kaden et al., 2016). In MS, MC-SMT seems to be able to distinguish chronic black-holes and thus, lesions with greater tissue damage from hyperintense T2 lesions (Bagnato et al., 2019, Bonet-Carne et al., 2019), and this approach can detect reductions in the apparent axon volume fraction in the spinal cord (SC) (By et al., 2018). Therefore, SMT-derived tissue features could be used as biomarkers to quantify the heterogeneous mechanisms involved in MS lesion pathogenesis *in vivo*.

Considering that MS lesions can display different degrees of damage, we hypothesized that the combination of several diffusion properties may be useful to characterize the severity of the changes in these lesions. Thus, measuring such variability could provide insights into the progression of disability and cognitive decline in patients with MS. Accordingly, the main aims of this study were to characterise MS lesions through macroscopic and microscopic diffusion information, and classify them in terms of the degree of damage, also determining the clinical relevance of the different types of lesions.

## 2 Materials and methods

### 2.1 Participants

We prospectively recruited a cohort of 59 MS patients at the MS Unit of the Hospital Clinic of Barcelona, 53 relapsing remitting (RR) and 6 secondary progressive (SP) patients according to 2010 McDonald criteria (Polman et al., 2011). Patients had to be relapse-free and free of corticosteroids in the month prior to testing. The Ethics Committee of the Hospital Clinic of Barcelona approved the study, and all participants provided their signed informed consent.

Demographic and clinical data were obtained from each participant, which included their score on the Expanded Disability Status Scale (EDSS) and its sub-scores for pyramidal, brainstem and cerebellum function (Kurtzke, 1983, Roxburgh et al., 2005). Their Multiple Sclerosis Severity Score (MSSS, Roxburgh et al., 2005) was also obtained and a cognitive assessment was performed using the Brief Repeatable Battery of neuropsychological tests (BRB-N, Rao et al., 1991). All raw values were transformed into z-scores according to published Spanish normative data (Sepulcre et al., 2006). The use of moderate-efficacy (interferon beta, glatiramer acetate, teriflunomide and dimethylfumarate) or high-efficacy (fingolimod, natalizumab, rituximab, ocrelizumab or cladribine) disease modifying therapies was registered.

### 2.2 Magnetic resonance imaging: Acquisition and processing

#### 2.2.1 Structural and diffusion magnetic resonance acquisition

MR images were acquired on a SIEMENS Magnetom Prisma^fit^ 3 T scanner with a 64-channel phased-array head/neck coil, and they included 3D-Magnetization Prepared Rapid Acquisition Gradient Echo (MPRAGE), 3D-T2 fluid-attenuated inversion recovery (FLAIR) and diffusion-weighted images (DWIs). Individual T1 3D-MPRAGE images had the following acquisition parameters: TR = 1800 ms; TE = 3.01 ms; TI = 900 ms; 240 sagittal slices with 0.94 mm isotropic voxel size and a 256 × 256 matrix size. The 3D-T2 FLAIR sequence parameters were: TR = 5000 ms; TE = 379 ms; TI = 1800 ms; 208 sagittal slices with 0.94 mm isotropic voxel size and a 256 × 256 matrix size. Multi-shell DWIs were acquired with: TR = 5400 ms; TE = 113 ms; parallel acceleration factor = 4; phase partial Fourier = 6/8; 100 contiguous axial slices at 1.5 mm isotropic voxel dimension; a 150 × 150 matrix size; b-values = 1000, 2000 and 3000 s/mm^2^ along 180 diffusion encoding directions; and 5b = 0 images. In addition, field map images were generated to estimate and correct susceptibility artifacts caused by field inhomogeneities (TE 1/TE 2 = 4.92/7.38 ms, with the same slice prescription, slice thickness and field of view as the multi-shell DWIs).

#### 2.2.2 Delineation mask and topography of MS lesions

MS lesions were manually delineated on the T1 3D-MPRAGE image, supported by a co-registered FLAIR image, using JIM software (Jim version 6.0 Xinapse System, http://www.xinapse.com/). We characterised each lesion independently through its cluster size and defined their location automatically. We established the lesions in which > 5% of their volume was in direct contact with the lateral ventricles as “periventricular lesions”, lesions with > 20% of their volume touching or within the cortex as “juxtacortical lesions”, and brainstem or cerebellar lesions as “infratentorial lesions” if >50% of their volume was placed in the brainstem or cerebellum. Finally, we considered the remaining lesions as “lesions located elsewhere in the deep WM” (Griffanti et al., 2018). Lesions smaller than 27 mm^3^ were excluded from the analysis (Filippi et al., 2019).

#### 2.2.3 Processing multi-shell diffusion MRI data

The diffusion imaging data was preprocessed using a combination of FSL and MRtrix software (Tournier et al., 2019). The low b-value was used to compute DTI metrics with FSL’s dtifit command by linear least-squares fitting method (Basser et al., 1994) and all the diffusion shells were employed to map the microstructural diffusivity (Kaden et al., 2016). Afterwards, we applied an inverse transformation matrix using boundary-based registration to place MS lesions into the diffusion space (Greve and Fischl, 2009). For each patient, the following measures were assessed for each individual MS lesion, and in the global NAWM: Location (periventricular, juxtacortical, infratentorial or deep WM); lesion volume; DTI-derived metrics (FA, mean diffusivity: MD, radial diffusivity: RD and axial diffusivity: AD); SMT microscopic diffusion coefficients (µFA, µMD, µRD and µAD); and multi-compartment SMT microscopic diffusion coefficients (intra-neurite volume fraction: ƒ_in__,_ intrinsic diffusivity: λ_diff__,_ extra-neurite transverse microscopic diffusivity: *v*_AD_ and extra-neurite microscopic mean diffusivity: *v*_MD_). The macroscopic and microscopic diffusion properties were selected to perform k-means cluster analysis to further extract the specific diffusion indices able to classify MS lesion types.

### 2.3 Statistical analysis

#### 2.3.1 Data-driven clustering of MS lesion types

We based the classification of MS lesions on diffusion imaging. We want to highlight here that clustering techniques may create artificial groups of data that may not be replicated in new data. To minimise this possibility, we only considered those sets of diffusion MRI measurements that led to clusters that were independently and consistently replicated in new data for periventricular, juxtacortical, brainstem, cerebellar and deep WM MS lesions, as defined by a prediction strength > 0.8 (Tibshirani and Walther, 2005). The “prediction strength” is a parameter proposed by Tibshirani and Walther that assesses how well the clustering obtained from one random half of the overall sample of lesions coincides with the clustering obtained from the other half of the sample. Specifically, for each set of diffusion tensor metrics and microscopic diffusion coefficients, we applied a standard k-means algorithm with k = 2 (i.e.: clustering the data into 2 groups) and performing a separate centroid-based classification for two random halves of the MS lesions, thereafter calculating the prediction strength. We are aware that there might be more than two types of lesions but for simplicity, we decided to only explore the two-type scenario - we understood that should there be three or more types of lesions, they might very well group as two main types. To avoid spurious results related to unfortunate divisions of the overall sample of lesions into two sets, we repeated this process 500 times and each time, the overall sample of lesions was divided randomly into two parts and the prediction strength was assessed. Subsequently, we averaged the corresponding 500 estimates of the prediction strength. Finally, to evaluate the significance of the prediction strength, we repeated these calculations after randomly assigning the diffusion characteristics of each lesion to other different lesions in order to create the distribution of prediction strengths under the null hypothesis (i.e.: that diffusion characteristics are not clustered). The resulting null distribution showed which prediction strengths could be expected by chance and thus, they allowed us to estimate the *p*-value.

#### 2.3.2 Relationships between clustering and clinical variables

Application of the selected clustering recognised two types of MS lesions, type A and B. We assessed whether the overall number or the volume of each type of lesion was correlated with the variables of disability. To do that, we fitted linear models with the clinical disability as the dependent variable, and the independent variables composed by the number or volume of each type of lesions, age and gender. Given that the residuals of some numeric variables may not follow a normal distribution, we found the statistical significance using the Freedman Lane permutation procedure, a common permutation test in neuroimaging studies due to its robustness to gross deviations of normality (Winkler et al., 2014). For binary variables of disability, we fitted logistic linear models, in which again the dependent variable was the variable of disability, and the independent variables were the number or volume of lesion types, age and gender. For the sake of comprehensiveness, we reassessed the correlations that proved to be statistically significant, on this occasion performing the analysis separately for the MS lesions at each brain location.

All the statistical analyses were carried out using the “fpc” (flexible procedures for clustering) package: https://cran.r-project.org/web/packages/fpc/index.html, implemented in the R platform (https://www.r-project.org/).

## 3 Results

Clinical, demographic and cognitive data was collected from the 59 MS patients included in the study (as summarised in Table 1), and the cohort had a mean age of 44.7 (±9.3) and 12.8 (±9.16) years of disease duration. Most patients were diagnosed with the RRMS form of the disease (90%).

**Table 1:** Demographic, clinical and cognitive data of the included participants.

	Multiple sclerosis patients n = 59
Age, years	44.7 (9.3)
Female, n (%)	37 (63)
MS type, n (%) Relapsing-remitting Secondary progressive	53 (90%) 6 (10%)
Disease modified treatment, n (%): Moderate-intensity therapy High-intensity therapy	40 (77) 12 (23)
Disease duration, years	12.8 (9.16)
EDSS score, median (range)	2.0 (0.0–7.5)
Cerebellar FS, median (range)	0 (0–4)
Pyramidal FS, median (range)	1 (0–5)
Brainstem FS, median (range)	0 (0–3)
MSSS, median (range)	2.28 (0.13–8.55)
Global cognitive z-score	−0.707 (1.011)
Visual memory z-score	−0.429 (1.071)
Verbal memory z-score	−1.045 (1.559)
Attention z-score	−0.492 (1.371)
Fluency z-score	−0.770 (1.125)

Continuous variables are given as the mean (standard deviation), except if defined otherwise. EDSS = Expanded Disability Status Scale; FS = Functional System; MSSS = Multiple Sclerosis Severity Score.

### 3.1 Characterization and classification of the MS lesions based on their diffusion properties

**Fig. 1:**
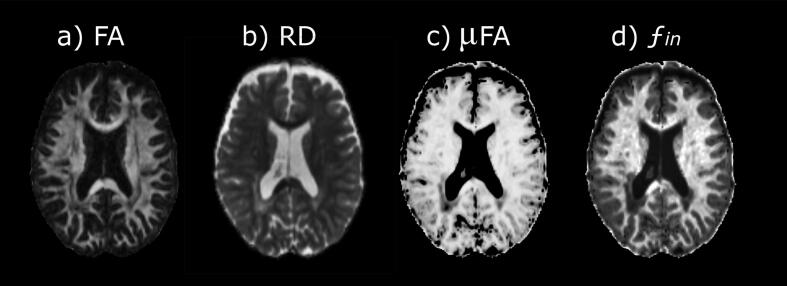
Diffusion measurements that classified lesions in two types. Diffusion maps from the DTI (a, b) and MC-SMT models (c, d) can distinguish A and B types MS lesions: FA = fractional anisotropy; RD = radial diffusivity; μFA = microscopic fractional anisotropy; ƒ_in_ = intra-neurite volume fraction.

We analysed 1,236 lesions in total, with a mean brain lesion volume of 11.37 (±15.30) cm^3^. We computed the mean DTI values and the microscopic properties of all lesions, both globally and at the distinct locations, as well as in the NAWM (Table 2). These diffusion imaging properties were weakly correlated, and we discarded the μAD and AD measures given their small variation in the lesions (38% of the values corresponded to the maximum value of this measure). Two sets of diffusion MRI indices at macro- and micro-scale were computed to identify different MS lesion profiles, the clustering of which showed prediction strengths>0.8, irrespective of the lesion localization. The first set were distinguished on the basis of the parameters FA, RD, μFA and ƒ_in_ (Fig. 1), while the second set was defined by the same parameters in the same directions, except that μFA was replaced by μRD (which was higher in B-type lesions). The groups of lesions defined by the two clusters were 99% identical and as such, we decided to limit the study to the first cluster as this had a slightly higher prediction strength (0.931).

**Table 2:** Description of diffusion properties in MS lesions and normal-appearing white matter.

	MS lesions						NAWM
	Whole brain	Periventricular	Juxtacortical	Brainstem	Cerebellum	Deep WM	
Total number of lesions analysed	1236	357	343	44	60	432	–
Mean lesion volume [cm^3^]	11.37 (15.30)	9.75 (14.90)	0.82 (0.59)	0.20 (0.20)	0.47 (1.14)	1.54 (5.02)	–
DTI-derived tensor metrics:							
FA	0.32 (0.11)	0.31 (0.12)	0.27 (0.10)	0.36 (0.08)	0.36 (0.12)	0.36 (0.11)	0.36 (0.04)
MD**	0.57 (0.09)	0.60 (0.08)	0.62 (0.08)	0.43 (0.07)	0.48 (0.08)	0.54 (0.08)	0.46 (0.03)
RD**	0.47 (0.10)	0.49 (0.09)	0.53 (0.09)	0.34 (0.07)	0.38 (0.09)	0.43 (0.09)	0.37 (0.04)
AD**	0.77 (0.11)	0.80 (0.10)	0.79 (0.11)	0.61 (0.10)	0.67 (0.09)	0.76 (0.11)	0.64 (0.03)
SMT microscopic diffusion tensor:							
μFA	0.83 (0.09)	0.78 (0.12)	0.82 (0.06)	0.93 (0.04)	0.91 (0.03)	0.87 (0.07)	0.90 (0.03)
μMD**	1.30 (0.19)	1.40 (0.23)	1.30 (0.13)	1.09 (0.08)	1.13 (0.08)	1.23 (0.14)	1.04 (0.06)
μRD**	0.44 (0.26)	0.60 (0.33)	0.47 (0.16)	0.18 (0.09)	0.23 (0.09)	0.35 (0.18)	0.23 (0.07)
μAD**	2.99 (0.11)	3.02 (0.07)	2.97 (0.14)	2.92 (0.12)	2.92 (0.12)	3.01 (0.09)	2.66 (0.07)
Multi-compartment microscopic diffusion coefficients:							
ƒ_in_	0.34 (0.13)	0.29 (0.10)	0.28 (0.09)	0.56 (0.02)	0.47 (0.15)	0.38 (0.13)	0.50 (0.06)
λ_diff_**	2.09 (0.35)	2.19 (0.32)	1.93 (0.31)	2.25 (0.40)	2.10 (0.48)	2.11 (0.34)	2.02 (0.16)
*V*_AD_**	1.35 (0.32)	1.54 (0.33)	1.40 (0.23)	0.92 (0.30)	1.10 (0.21)	1.27 (0.28)	0.91 (0.13)
*V*_MD_**	1.60 (0.28)	1.75 (0.30)	1.60 (0.23)	1.36 (0.18)	1.40 (0.20)	1.55 (0.24)	1.28 (0.10)

Continuous variables are given as the mean (standard deviation). The numbers are the lesion counts in the first row and the mean metrics across the lesions in the other rows. FA = Fractional anisotropy; MD = Mean diffusivity; RD = Radial diffusivity; AD = Axial diffusivity; ƒ_in_ = intra-neurite volume fraction; λ_diff_ = intrinsic diffusivity; *V*_AD_ = extra-neurite transverse microscopic diffusivity; *V*_MD_ = extra-neurite microscopic mean diffusivity. **units mm^2^/s × 10^-3^

**Fig. 2:**
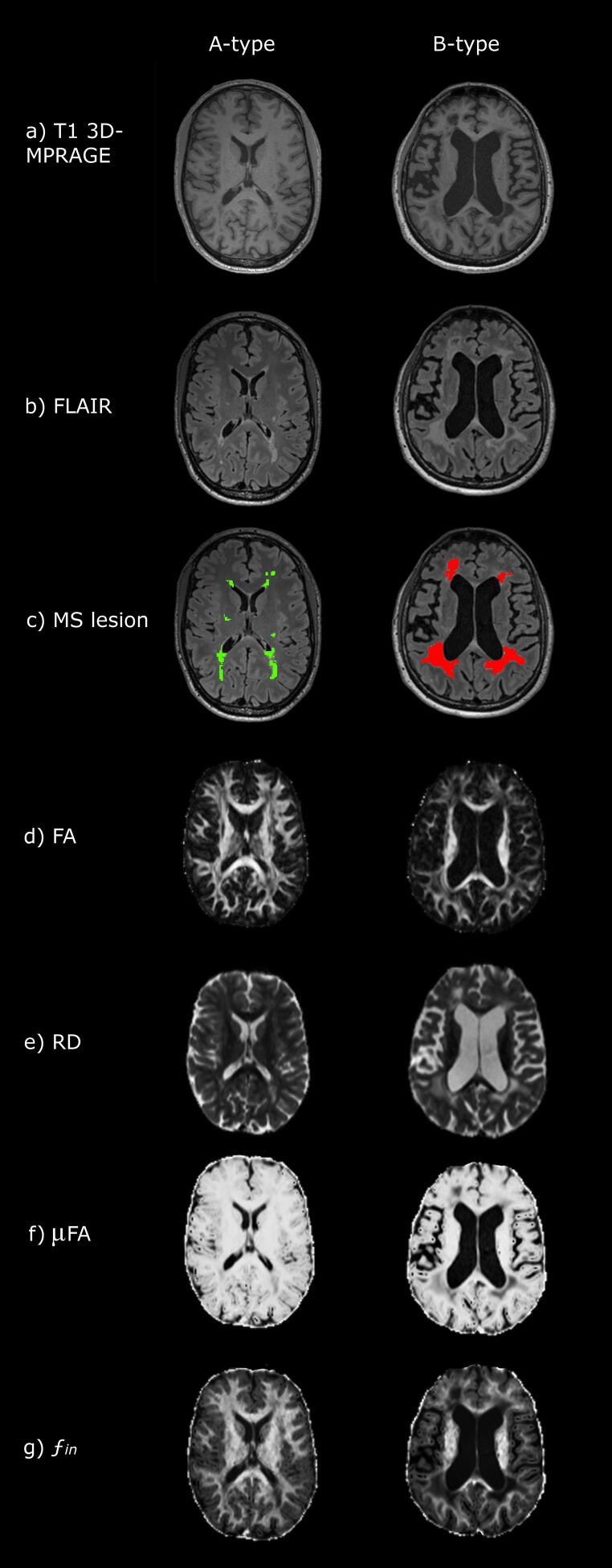
Example of two patients that presented a predominant lesion type. Most lesions were classified as A-type (in green) in the patient in the left column, while the majority of lesions were B-type (in red) in the patient in the right column: FA = fractional anisotropy; RD = radial diffusivity; μFA = microscopic fractional anisotropy; ƒ_in_ = intra-neurite volume fraction. (For interpretation of the references to colour in this figure legend, the reader is referred to the web version of this article.)

**Supplementary figure 1:**
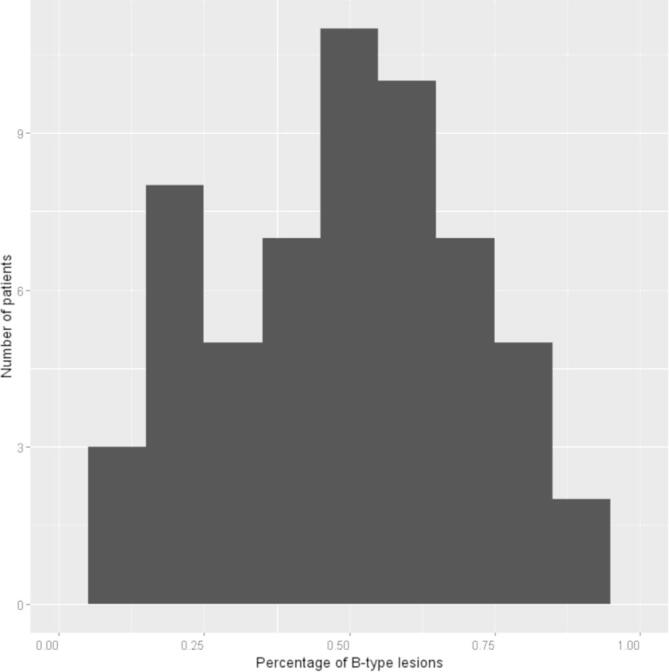


**Supplementary figure 2:**
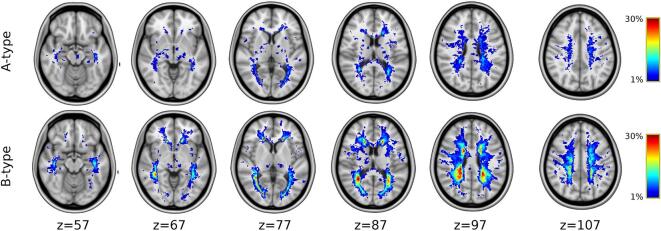


Compared to A-type lesions, B-type lesions had a lower FA, μFA and ƒ_in_, yet a higher RD, irrespective of the lesion location (P < 0.001, Table 3 and Supplementary Table 1). Moreover, the lesion number of both types were similar at all the locations, except at the juxtacortical regions where the B-type lesions predominated (P < 0.001). Most patients had both types of lesions, with between 40 and 70% of B-type lesions (Supplementary Fig. 1). Moreover, 27% of patients had 80% or more of one specific type of plaque, with a predominance of A-type lesions in 15% of the subjects, while 12% had mostly B-type lesions (see Fig. 2). However, there was no correlation between the overall number of A and B type lesions. By contrast, 64 and 63% of the lesion volume corresponded to B-type lesions in the whole brain and periventricular areas, respectively, suggesting that the plaques of this type were larger (Table 3, Supplementary Fig. 2). The proportional volumes of B-type lesions but not their number was higher in the SPMS patients (mean percentage 90%) than in the RRMS patients (mean percentage 61%: 95% CI −0.48 to −0.10; P < 0.01).

**Table 3:** Description of the B-type lesions compared to A-type lesions.

	Whole brain	Periventricular	Juxtacortical	Brainstem	Cerebellum	Deep WM
Prediction strength of lesion classification	0.931	0.822	0.820	0.925	0.820	0.824
Percentage of B-type lesions	52%	54%	60% (P < 0.001)	50%	53%	46%
Percentage of B-type lesions volume	64% (P < 0.001)	63% (P < 0.001)	50%	48%	50%	55%
Differences between DTI-derived tensor metrics and microstructural diffusion properties comparing MS lesion types						
ΔFA	−0.15	−0.16	−0.13	−0.10	−0.18	−0.14
ΔRD**	0.17	0.15	0.15	0.12	0.15	0.15
ΔμFA	−0.12	−0.14	−0.14	−0.05	−0.05	−0.09
Δƒ_in_	−0.20	−0.16	−0.14	−0.27	−0.22	−0.19

Continuous variables are given as the mean (standard deviation). All diffusion metrics showed significant differences (P < 0.001) between A and B types lesions: FA = fractional anisotropy; RD = radial diffusivity; μFA = microscopic fractional anisotropy; ƒ_in_ = intra-neurite volume fraction. Δ = delta/difference. **units of mm^2^/s × 10^-3^.

### 3.2 Association between MS lesion type and the clinical outcome

At the patient-level, we detected several significant correlations between the overall number of B-type lesions and the clinical variables (P < 0.05 controlling for the effect of age and gender: Table 4). By contrast, we did not find any significant correlation between A-type lesions and the clinical data. Thus, a higher number of B-type lesions was associated with a higher MSSS, worse cerebellar function and worse cognition (Bonferroni-corrected P threshold = 0.004). Juxtacortical and cerebellar lesions had the strongest correlation values. However, we failed to detect significant correlations with clinical data when the number of periventricular B-type lesions was considered.

**Table 4:** Association between number of B-type lesions and the clinical variables, controlling for the effects of age and gender.

	Global	Periventricular	Juxtacortical	Brainstem	Cerebellar	Deep WM
MSSS	Positive (P = 0.004)*	n.s	Positive (P = 0.006)	n.s	Positive (P = 0.001)*	Positive (P = 0.042)
EDSS	Positive (P = 0.015)	n.s	Positive (P = 0.025)	n.s	Positive (P = 0.001)*	Positive (P = 0.029)
Cerebellar functional system	Positive (P < 0.001)*	n.s	Positive (P = 0.001)*	n.s	Positive (P < 0.001)*	Positive (P = 0.003)*
High-efficacy therapy	Positive (P = 0.008)	n.s	Positive (P = 0.017)	n.s	Positive (P = 0.017)	Positive (P = 0.016)
*Neuropsychological test battery*						
Global cognitive score	Negative (P < 0.001)*	n.s	Negative (P < 0.001)*	Negative (P = 0.001)*	Negative (P < 0.001)*	Negative (P = 0.002)*
zAttention	Negative (P < 0.001)*	n.s	Negative (P = 0.001)*	Negative (P = 0.005)	Negative (P = 0.001)*	Negative (P = 0.002)*
zFluency	Negative (P = 0.033)	n.s	n.s.	Negative (P = 0.014)	Negative (P = 0.007)	n.s
zVerbal memory	Negative (P = 0.001)*	n.s	Negative (P = 0.001)*	Negative (P = 0.016)	Negative (P = 0.004)	Negative (P = 0.003)*

MSSS = Multiple Sclerosis Severity Score; EDSS = Expanded Disability Status Scale; n.s. = not statistically significant; *, significant after a Bonferroni correction.

In terms of lesion volume, the volume of B-type lesions was correlated with cerebellar function and cognitive disability. In particular, stronger correlations with clinical disability were found for periventricular lesions (Table 5). However, there were no significant correlations with EDSS, brainstem and pyramidal functional systems, verbal fluency, visual memory deficits and the type of treatment after a Bonferroni correction.

**Table 5:** Associations between the volume of B-type lesions and the clinical variables controlling for the effects of age and gender.

	Whole brain	Periventricular	Juxtacortical	Brainstem	Cerebellar	Deep WM
EDSS	Positive (P = 0.021)	Positive (P = 0.025)	n.s	n.s	n.s	n.s
Cerebellar functional system	Positive (P < 0.001)*	Positive (P < 0.001)*	Positive (P = 0.014)	n.s	Positive (P = 0.001)	n.s
Brainstem functional system	Positive (P = 0.016)	Positive (P = 0.014)	n.s.	n.s.	n.s.	n.s.
Pyramidal functional system	Positive (P = 0.010)	Positive (P = 0.008)	n.s.	n.s.	n.s.	n.s.
High-efficacy therapy	Positive (P = 0.022)	Positive (P = 0.022)	n.s	n.s	n.s	n.s
*Neuropsychological test battery*						
Global cognitive score	Negative (P < 0.001)*	Negative (P < 0.001)*	Negative (P = 0.029)	Negative (P = 0.003)	n.s	n.s
zAttention	Negative (P < 0.001)*	Negative (P < 0.001)*	Negative (P = 0.034)	Negative (P = 0.037)	n.s.	n.s
zFluency	Negative (P = 0.015)	Negative (P = 0.019)	n.s	n.s	n.s	n.s
zVerbal memory	Negative (P < 0.001)*	Negative (P < 0.001)*	n.s	Negative (P = 0.019)	n.s	Negative (P = 0.016)
zVisual memory	Negative (P = 0.012)	Negative (P = 0.009)	n.s	n.s	n.s	n.s

EDSS = Expanded Disability Status Scale; n.s. = not statistically significant; *, significant after a Bonferroni correction.

## 4 Discussion

In this study, we demonstrate that MS lesions can be classified into two types based on the severity of the changes in terms of macroscopic DTI parameters and microscopic diffusion properties. We found that most patients had both types of lesions, although in nearly a quarter of the cohort there was a clear predominance towards a given lesion type. B-type lesions are thought to present more severe tissue damage, and in terms of number and volume, the study demonstrates that their presence is related to a worse clinical evolution. Specifically, a larger number of B-type lesions in the juxtacortical, cerebellar and deep WM areas was more strongly associated with disability, as was a larger volume of these lesions in periventricular regions. All in all, the results support the usefulness of diffusion MRI to obtain information *in vivo* on the heterogeneity of the pathological changes in MS plaques.

Our findings indicate that the combination of two diffusion-based models, DTI (FA and RD) and MC-SMT (μFA and ƒ_in_), which can capture how water moves in the tissue over distinct timescales, enables two distinct types of MS lesions to be classified with high predictive value. Lesions with larger modifications in diffusion imaging properties are crucial to characterize the two MS lesion types (A-type lesions show higher FA, μFA and ƒ_in_, and smaller RD values; while B-type lesions display lower FA, μFA and ƒ_in_, and higher RD values on Supplementary Fig. 3). B-type lesions are thought to be associated with more severe demyelination and axonal damage (Yu et al., 2019). Therefore, the classification proposed would provide information regarding inflammatory destruction or the ability for neurorepair in a given patient, potentially representing a useful biomarker for phase II clinical trials.

In previous studies, focal MS lesions display very heterogeneous DTI abnormalities, with a persistent decrease in FA values and an increase in the other diffusion coefficients compared to the NAWM (Inglese and Bester, 2010). FA values preferentially reflect changes in axon density, whilst RD is a measure sensitive to myelin injury (Beaulieu, 2002) . However, these diffusion features alone are not sufficiently specific to estimate the severity of damage. Moreover, their association with clinical disability is mild to moderate due to the large variability of DTI indices and the complex processes lesioned tissues undergo (Filippi et al., 2001). Conversely, μFA and ƒ_in_ provide information regarding more specific features at the microstructural level, depicting restricted anisotropic diffusion into the intracellular water domain (Kaden et al., 2008). Accordingly, despite the MC-SMT model does not allow the quantification of non-monoexponential behavior to describe the deviation of diffusion displacement from the Gaussian profile specifically (Jensen et al., 2005), a significant decrease of μFA and ƒ_in_ have been demonstrated for different degrees of brain and SC tissue damage in MS compared with normal WM tissue (By et al., 2018, Lakhani et al., 2020). Furthermore, such microscopic features seem to be able to distinguish MS lesions with more axonal damage from the lesions that are hyperintense in T2-weighted sequences (Bagnato et al., 2019, Bonet-Carne et al., 2019), identified as black-holes in T1-spin echo sequences (van Walderveen et al., 1998). When compared with the observation of black holes, the use of quantitative diffusion metrics increases the accuracy and reproducibility of the results. Thus, our findings highlight the complementarity of DTI and SC-SMT metrics to define the characteristics of MS lesions.

The proportion of A and B type lesions was similar across the brain, except in juxtacortical areas where B-type lesions predominate. In periventricular regions, most of the lesion volume corresponds to B-type lesions, and such regional differences could reflect the nature of MS lesions in terms of their formation and evolution. This hypothesis is supported by the predominance of B-type lesions in SPMS patients (mean = 90%). Nevertheless, further longitudinal studies will be required to decipher the chronicity of those lesions and to assess whether they are related to slowly expanding plaques.

Previous studies showed that focal MS lesions, a hallmark of the disease, are weakly correlated with clinical disability (Barkhof, 2002) and disease severity (Mostert et al., 2010). However, our findings demonstrate that the number and volume of specific B-type lesions were strongly associated with a more severe disease evolution (correlation coefficients between 0.4 and 0.67), with a worse physical (mainly related to cerebellar functions) and cognitive disability. The lack of correlation with the EDSS after correcting for multiple comparisons could be influenced by the strong influence of SC integrity on the EDSS (Rocca et al., 2017), a fact that was not assessed here. Specifically, the number and volume of B-type lesions in juxtacortical and cerebellar areas, and their volume in periventricular regions, were the features that were most strongly correlated with disease evolution and disability. Indeed, periventricular damage may affect large white matter tracts, such as the cingulum and frontoparietal connections, potentially contributing to the cognitive deficits in patients with MS (Tiemann et al., 2009, Solana et al., 2018). Previous studies reported results consistent with the present findings, correlating brain lesion with a worsening in clinical disability, particularly for T1 hypointense lesions (Giorgio et al., 2014). Together, the presence of lesions with larger diffusion changes could reflect a destructive pattern of chronically demyelinated axons and more neuroaxonal damage, which is related to more severe disease evolution.

This study has several limitations that should be considered for future research. First, our findings should be validated through histological studies to characterize the underlying tissue changes in the A and B type lesions, and their correspondence with active, chronic or chronic active lesions. Second, diffusion metrics are highly dependent on acquisition and scanner parameters, although they are very reproducible in scan-rescan experiments (By et al., 2018). Consequently, it is important to harmonize the techniques for clinical trials that focus on different sites and protocols (Fortin et al., 2017). Finally, we did not evaluate the specific microscopic and macroscopic changes in new T1-enhancing lesions, in black holes or over time, and thus, longitudinal studies would be useful to understand the MS temporal evolution and their predictive value in a prospective manner.

## 5 Conclusions

Microscopic features of the intracellular water domain (μFA and ƒ_in_) and macroscopic DTI-derived metrics (FA, RD) together contribute to define the amount of damage within MS lesions. In turn, these features provide a specific pattern of lesion severity that helps understand the mechanisms underlying clinical disability and cognitive impairment in MS patients. Accordingly, the classification of lesion types has the potential to ensure MS patients receive more specific and better-targeted therapies.

## CRediT authorship contribution statement

**Eloy Martínez-Heras:** Conceptualization, Methodology, Software, Validation, Investigation, Writing - original draft, Visualization, Supervision. **Elisabeth Solana:** Conceptualization, Methodology, Software, Validation, Investigation, Writing - original draft, Visualization, Supervision. **Ferran Prados:** Formal analysis, Software, Supervision. **Magí Andorrà:** . **Aleix Solanes:** Formal analysis, Software, Supervision. **Elisabet López-Soley:** Writing - review & editing. **Carmen Montejo:** Writing - review & editing. **Irene Pulido-Valdeolivas:** Writing - review & editing. **Salut Alba-Arbalat:** Writing - review & editing. **Nuria Sola-Valls:** Writing - review & editing. **Maria Sepúlveda:** Writing - review & editing. **Yolanda Blanco:** Writing - review & editing. **Albert Saiz:** Resources, Supervision, Writing - review & editing, Funding acquisition. **Joaquim Radua:** Formal analysis, Software, Supervision, Writing - review & editing. **Sara Llufriu:** Conceptualization, Investigation, Resources, Writing - review & editing, Supervision, Funding acquisition.

## Declaration of Competing Interest

The authors declare that they have no known competing financial interests or personal relationships that could have appeared to influence the work reported in this paper.

## Figures and Tables

**Supplementary figure 3:**