# Supplementary material for: Characterization of multiple sclerosis lesions with distinct clinical correlates through quantitative diffusion MRI
Source: Neuroimage Clin. 2020 Sep 9;28:102411. doi: 10.1016/j.nicl.2020.102411 (PMC7502564; doi:10.1016/j.nicl.2020.102411)
Supplement: Supplementary data 1 [file mmc1.docx]

Supplementary Table 1. Diffusion properties of each type of MS lesions for each location.

|  | Whole brain | | Periventricular | | Juxtacortical | | Brainstem | | Cerebellum | | Deep WM | |
| --- | --- | --- | --- | --- | --- | --- | --- | --- | --- | --- | --- | --- |
| Measure | A-type | B-type | A-type | B-type | A-type | B-type | A-type | B-type | A-type | B-type | A-type | B-type |
| FA | 0.4060  (0.098) | 0.254  (0.074) | 0.397  (0.11) | 0.240  (0.07) | 0.348  (0.09) | 0.218  (0.06) | 0.411  (0.08) | 0.313  (0.05) | 0.459  (0.10) | 0.278  (0.07) | 0.427  (0.09) | 0.287  (0.07) |
| RD** | 0.3790  (0.061) | 0.5490  (0.062) | 0.416  (0.06) | 0.565  (0.05) | 0.440  (0.05) | 0.587  (0.06) | 0.285  (0.04) | 0.402  (0.03) | 0.302  (0.05) | 0.448  (0.05) | 0.365  (0.05) | 0.514  (0.05) |
| μFA | 0.9010  (0.040) | 0.7790  (0.094) | 0.853  (0.06) | 0.709  (0.12) | 0.872  (0.03) | 0.786  (0.05) | 0.958  (0.02) | 0.909  (0.03) | 0.940  (0.02) | 0.891  (0.03) | 0.915  (0.03) | 0.821  (0.07) |
| ƒ_in_ | 0.4485  (0.115) | 0.2453  (0.057) | 0.376  (0.08) | 0.221  (0.05) | 0.356  (0.07) | 0.221  (0.04) | 0.691  (0.14) | 0.420  (0.06) | 0.585  (0.12) | 0.362  (0.08) | 0.472  (0.10) | 0.279  (0.06) |

**units of mm^2^/s x 10^-3^

Variables are given as mean +- standard deviation.
